# Organizational transitions in awareness: a framework of access, coordination, and regulation

**DOI:** 10.3389/fpsyg.2026.1892596

**Published:** 2026-07-29

**Authors:** Alison King, Darryl Gray

**Affiliations:** 1 Independent Researcher, Toowoomba, QLD, Australia; 2 Independent Researcher, Brisbane, QLD, Australia

**Keywords:** anesthesia, awareness processes, biological regulation, consciousness, integrated experiential awareness, metacognition, ordered vulnerability, recursive self-modeling

## Abstract

Consciousness research often uses overlapping terms such as consciousness, awareness, conscious access, subjective experience, reportability, selfhood, and metacognition. This conceptual compression can make it difficult to distinguish different awareness-related processes or to identify which capacities are preserved, disrupted, or restored across altered states. This paper proposes an organizational framework for distinguishing awareness processes in terms of constrained access, representational coordination, adaptive regulation, and recursive self-modeling. The framework distinguishes three related but partially separable organizational conditions: biological regulation, integrated experiential awareness, and recursive self-modeling. Here, partially separable means that these organizational conditions may overlap and interact while nevertheless exhibiting different patterns of degradation, persistence, or recovery under perturbation. Biological regulation refers to adaptive control and responsiveness that need not involve stable experiential organization. Integrated experiential awareness refers to first-order experiential organization in which sensory, interoceptive, affective, and contextual information is coordinated and stabilized across time. Recursive self-modeling refers to the capacity of a system to represent aspects of its own internal, evaluative, or behavioral states across time. The central hypothesis is that these conditions may show ordered vulnerability across perturbation and recovery. Recursive self-modeling should often be more vulnerable than integrated first-order experiential organization under conditions such as sleep onset, anesthesia, dissociation, depersonalization, neurological disruption, and other altered states, and should often re-emerge later during recovery. This prediction does not require sharp thresholds or fixed natural boundaries. Instead, the proposed conditions are treated as graded transition zones within a multidimensional organizational landscape. The framework is positioned as an organizational crosswalk rather than a replacement for existing theories. Its aim is to clarify relationships between biological regulation, experiential organization, metacognitive accessibility, and recursive self-related processing while generating testable predictions for consciousness research.

## 1 Introduction

Consciousness science continues to face persistent conceptual and empirical difficulties despite major advances in neuroscience, cognitive science, computational modeling, and the study of altered states. One recurring difficulty is that terms such as consciousness, awareness, subjective experience, conscious access, reportability, selfhood, and metacognition are often used in overlapping ways despite referring to partially distinct phenomena.

In some contexts, consciousness denotes phenomenal experience. In others, it refers to wakefulness, behavioral responsiveness, perceptual accessibility, verbal report, metacognitive monitoring, or higher-order self-referential processing. This conceptual compression complicates attempts to identify clear empirical targets and compare theoretical approaches across consciousness science.

Existing theories already emphasize different aspects of this problem. Integrated Information Theory foregrounds informational integration and differentiation (Tononi, 2004; Tononi et al., 2016). Global Workspace and Global Neuronal Workspace approaches emphasize large-scale accessibility and distributed coordination (Baars, 1988; Dehaene and Changeux, 2011). Predictive processing and active inference emphasize adaptive modeling and regulation under uncertainty (Friston, 2010; Seth et al., 2012). Higher-order, metacognitive, and self-model approaches emphasize reflective accessibility, confidence monitoring, and self-related representation (Lau and Rosenthal, 2011; Metzinger, 2003).

Recent empirical work has also suggested that conscious report may depend on temporally extended integrative accumulation dynamics rather than isolated sensory activation alone (Lozito et al., 2026). This is consistent with the present paper's emphasis on graded transition zones rather than sharp boundaries between awareness-related conditions.

The present paper does not propose a replacement theory of consciousness. By a replacement theory, we mean a theory intended to supersede or replace existing theories of consciousness rather than clarify, relate, or integrate their respective domains. Instead, it develops an organizational framework for distinguishing awareness processes in terms of constrained access, representational coordination, adaptive regulation, and recursive self-modeling.

The framework distinguishes three related but partially separable organizational conditions: biological regulation, integrated experiential awareness, and recursive self-modeling. Biological regulation refers to adaptive control and responsiveness that need not involve stable experiential organization. Integrated experiential awareness refers to first-order experiential organization in which sensory, interoceptive, and contextual information is coordinated and stabilized across time. Recursive self-modeling refers to the capacity of a system to represent aspects of its own internal, evaluative, or behavioral states across time.

The central empirical claim is an ordered vulnerability hypothesis: recursive self-modeling should often be more vulnerable than integrated first-order experiential organization under perturbation and should often re-emerge later during recovery. This prediction does not require the proposed conditions to be sharp natural boundaries. Rather, they are treated as graded transition zones within a multidimensional organizational landscape.

Central to the framework is the principle of structural dependence: awareness processes vary according to underlying organizational structure, including representational coordination, temporal stability, constrained access, adaptive regulation, and recursive accessibility. Under this interpretation, altered states and developmental or clinical transitions should produce partially dissociable changes across experiential organization, metacognitive accessibility, self-reference, behavioral flexibility, and temporal continuity rather than uniform loss of a single indivisible property.

The contribution of the present framework is therefore modest but testable. It does not attempt to resolve consciousness as a singular phenomenon or explain why subjective experience exists at all. Instead, it proposes a structured way of distinguishing first-order experiential organization from recursive self-modeling and of testing their relationship across changing states.

**Figure 1:**
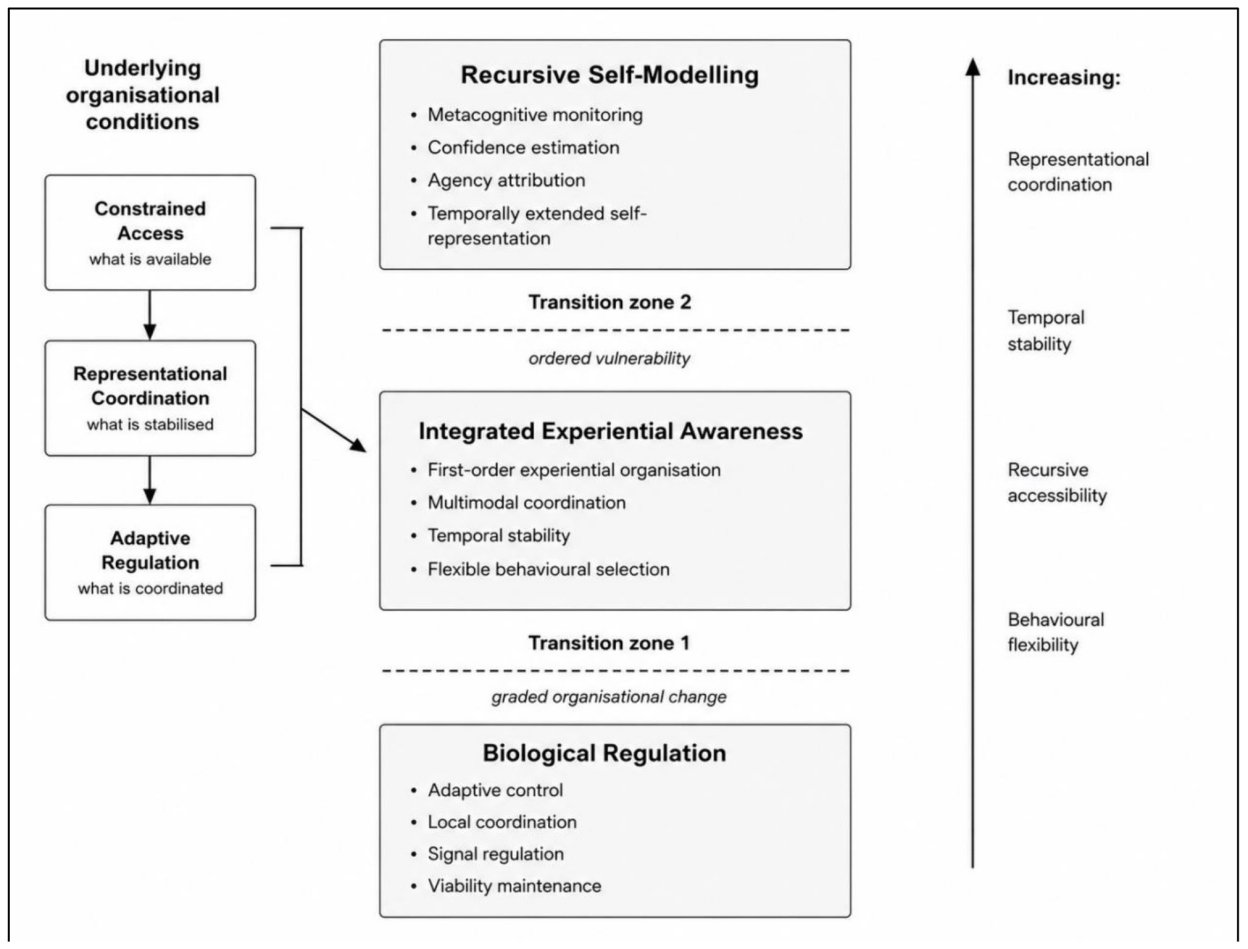
Organizational Conditions and Transition Zones in Awareness-Related Processing. The framework distinguishes three partially separable organizational conditions: biological regulation, integrated experiential awareness, and recursive self-modeling. Transition zones are interpreted as graded organizational changes rather than sharp categorical boundaries. Biological regulation supports adaptive control and viability maintenance. Integrated experiential awareness involves temporally stabilized first-order experiential organization. Recursive self-modeling involves metacognitive accessibility, confidence estimation, agency attribution, and temporally extended self-representation. The framework predicts that recursive self-modeling will generally show greater vulnerability under perturbation and slower recovery than integrated experiential awareness.

Figure 1 summarizes the organizational conditions and transition zones proposed in the framework.

The sections that follow define the organizational conditions, clarify the ordered vulnerability hypothesis, situate the framework in relation to existing theories, and outline empirical pathways for investigating dissociation, degradation, and recovery across sleep, anesthesia, disorders of consciousness, altered states, developmental cognition, comparative cognition, and boundary cases.

## 2 Organizational conditions and awareness processes

These organizational conditions are not proposed as rigid stages, metaphysical categories, or sharply defined natural kinds. Rather, they are proposed as useful distinctions for separating processes that are often compressed under broad terms such as consciousness or awareness.

The framework treats these conditions as graded and partially overlapping. A system may preserve some awareness-related processes while others degrade, fluctuate, or recover at different rates. This is especially relevant in altered states, developmental transitions, disorders of consciousness, and conditions involving disruption to self-related processing.

### 2.1 Biological regulation

Biological regulation refers to adaptive control and responsiveness that need not involve stable experiential organization or recursive self-modeling. Biological systems may regulate behavior and maintain viability through:

- homeostatic control,
- autonomic regulation,
- sensorimotor responsiveness,
- chemotaxis,
- tropistic behavior,
- and anticipatory physiological adjustment.

These processes may be adaptive, flexible, and uncertainty-sensitive without necessarily implying integrated experiential awareness. For this reason, the framework does not treat adaptive regulation alone as sufficient evidence for awareness.

Biological regulation therefore provides the organizational background from which more complex awareness processes may emerge, but it is not treated as equivalent to experiential organization.

### 2.2 Integrated experiential awareness

Integrated experiential awareness refers to first-order experiential organization in which sensory, interoceptive, affective, and contextual information is coordinated and stabilized across time. This condition is proposed to support:

- coherent perceptual content,
- multimodal world/body organization,
- flexible behavioral selection,
- and context-sensitive interaction beyond immediate stimulus-response coupling.

Integrated experiential awareness is not defined solely by reportability, verbal access, or explicit introspection. The framework allows that experiential organization may persist under conditions in which reflective monitoring, self-reference, or behavioral report are reduced or absent.

Dreaming provides one useful example. Ordinary dreams may preserve vivid experiential organization while metacognitive accessibility, autobiographical continuity, and reflective self-monitoring remain unstable or reduced.

The framework does not claim that any single marker is sufficient to establish integrated experiential awareness. Large-scale integration, recurrent coordination, effective connectivity, signal complexity, behavioral flexibility, and reportability may each provide useful evidence, but none is treated as identical with experiential organization itself.

### 2.3 Recursive self-modeling

Recursive self-modeling refers to the capacity of a system to represent aspects of its own internal, evaluative, or behavioral states across time. This condition may support:

- metacognitive accessibility,
- confidence estimation,
- error monitoring,
- agency attribution,
- self-evaluation,
- and temporally extended self-related continuity.

Here, agency attribution refers to the process by which a system represents an action, intention, or outcome as self-generated, self-related, other-generated, or externally caused. This is distinct from agency itself, which refers more generally to the capacity to initiate or control action.

Importantly, recursive self-modeling is not treated as a single all-or-none capacity. Core metacognitive self-monitoring may dissociate from more extended forms of autobiographical or narrative selfhood. Similarly, minimal self-related processing may dissociate from explicit reflective self-representation.

This distinction is important because altered states may preserve integrated experiential organization while selectively disrupting metacognitive accessibility, agency attribution, autobiographical continuity, or stable narrative identity.

Within the present framework, recursive self-modeling is therefore proposed to depend on more demanding organizational conditions than integrated experiential awareness alone.

### 2.4 Operational distinctions

The framework distinguishes between organizational conditions, evidence consistent with those conditions, and indicators that are not sufficient on their own. This distinction is important because measures commonly used in consciousness research may reflect correlates, enabling conditions, or behavioral outputs rather than awareness processes themselves.

Table 1 summarizes these operational distinctions. The central prediction of the framework is not that all awareness processes rise and fall together, but that these organizational conditions may show partially ordered patterns of preservation, degradation, and recovery across changing biological and cognitive states.

**Table 1:** Operational distinctions between organizational conditions.

Organizational condition	Core definition	Evidence consistent with the condition	Not sufficient on its own
Biological regulation	Adaptive control processes maintaining viability and behavioral adjustment without requiring stable experiential organization or recursive self-modeling	Homeostatic regulation, sensorimotor adaptation, anticipatory adjustment, uncertainty-sensitive control	Mere responsiveness, behavioral adaptation, or predictive regulation alone
Integrated experiential awareness	Temporally stabilized first-order experiential organization supporting coherent multimodal content and flexible behavioral coordination across time	Cross-domain coordination, recurrent processing, sustained perceptual organization, context-sensitive behavioral flexibility	Complexity measures alone, effective connectivity alone, reportability alone, or behavioral flexibility alone
Recursive self-modeling	Recursive accessibility to internal representational, evaluative, or behavioral states supporting metacognitive monitoring and temporally extended self-related regulation	Confidence estimation, metacognitive sensitivity, agency monitoring, explicit self-evaluation, temporally extended self-reference	Own-name effects alone, self-prioritization alone, mirror recognition alone, or autobiographical memory alone

## 3 Transition zones and ordered vulnerability

The present framework treats the movement between biological regulation, integrated experiential awareness, and recursive self-modeling as graded transition zones rather than sharp thresholds. These transitions are not proposed as fixed natural boundaries. They are proposed as useful ways of describing changes in organizational coordination, temporal stability, behavioral flexibility, and recursive accessibility.

The central empirical claim is an ordered vulnerability hypothesis: recursive self-modeling should often be more vulnerable than integrated experiential awareness under perturbation and should often re-emerge later during recovery.

This does not imply a universal sequence across every state or system. Rather, it predicts a broad tendency: organizational conditions supporting recursive self-modeling should generally be more fragile because they depend on additional forms of metacognitive accessibility, self-related continuity, and temporal stability.

### 3.1 From biological regulation to integrated experiential awareness

The first transition zone concerns the movement from biological regulation to integrated experiential awareness.

Biological regulation may already involve adaptive responsiveness, anticipatory adjustment, and uncertainty-sensitive control. However, integrated experiential awareness is proposed to require a further organizational condition: first-order experiential organization that is coordinated and stabilized across time. This transition may involve increasing:

- cross-domain coordination,
- recurrent processing,
- temporal stability,
- multimodal organization,
- and flexible behavioral selection.

Importantly, the framework does not claim that any single marker defines this transition. Complexity measures, effective connectivity, recurrent coordination, behavioral flexibility, and reportability may each provide relevant evidence, but none is treated as sufficient on its own.

The transition is therefore best understood as a shift toward temporally stabilized experiential organization rather than as the appearance of one isolated mechanism or behavioral marker.

### 3.2 From integrated experiential awareness to recursive self-modeling

The second transition zone concerns the movement from integrated experiential awareness to recursive self-modeling.

In this transition, a system becomes capable of representing aspects of its own internal, evaluative, or behavioral states across time. This may support confidence estimation, error monitoring, agency attribution, self-evaluation, and temporally extended self-related continuity.

This transition is not treated as a single all-or-none event. Core metacognitive self-monitoring may emerge separately from more extended autobiographical or narrative selfhood. Minimal self-related processing may also dissociate from explicit reflective self-representation.

The framework therefore treats recursive self-modeling as a layered organizational condition rather than a single unified capacity.

This distinction is important because some states may preserve vivid experiential organization while disrupting metacognitive accessibility, agency attribution, autobiographical continuity, or stable narrative identity. Ordinary dreaming, dissociation, depersonalization, anesthetic transitions, and neurological disruption may each provide examples of this kind of partial dissociation.

### 3.3 Ordered vulnerability and recovery

The ordered vulnerability hypothesis predicts that recursive self-modeling should often be more fragile than integrated experiential awareness.

Under perturbation, recursive self-modeling is expected to destabilize earlier, degrade more rapidly, and recover later than integrated first-order experiential organization.

This prediction follows from the additional organizational demands required for recursive self-modeling. Unlike integrated experiential awareness, recursive self-modeling requires access to internal states as objects of monitoring, evaluation, and regulation across time.

The framework therefore predicts that some altered states should preserve aspects of experiential organization while selectively impairing metacognitive accessibility, agency attribution, confidence monitoring, or autobiographical continuity. During recovery, the reverse pattern may occur: first-order experiential organization may return before stable recursive self-modeling is restored.

**Figure 2:**
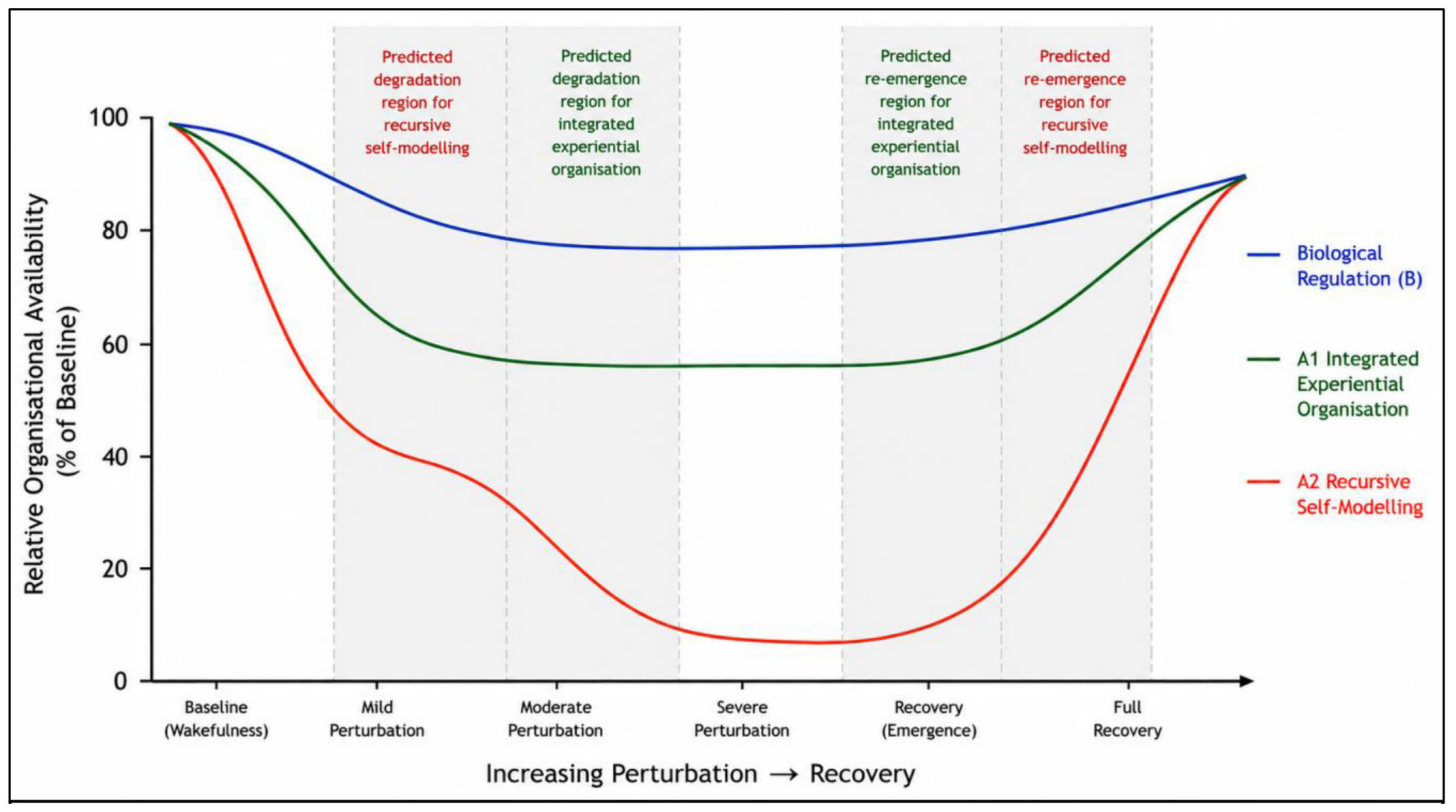
Hypothetical ordered vulnerability across perturbation and recovery. Biological regulation is predicted to remain comparatively preserved across perturbation. Integrated experiential organization (A1) is predicted to degrade under increasing perturbation but re-emerge before recursive self-modeling (A2). Recursive self-modeling is predicted to show greater vulnerability and slower recovery because it depends on additional organizational conditions, including metacognitive accessibility, agency attribution, and temporally extended self-related continuity. The shaded areas indicate predicted regions of degradation and re-emergence within broader transition zones rather than fixed thresholds. The figure is illustrative and does not represent measured data or imply a universal sequence across all individuals, states, or conditions.

This prediction is probabilistic rather than absolute. Figure 2 illustrates the ordered vulnerability hypothesis as a general organizational principle rather than a fixed sequence. The framework does not claim that all altered states must follow the same trajectory, only that recursive self-modeling will often show greater organizational vulnerability under conditions of reduced coordination, instability, or perturbation. Predicted transitions are therefore best understood as occurring across broader transition zones rather than at sharp categorical boundaries.

### 3.4 Boundary conditions and potential exceptions

The ordered vulnerability hypothesis is probabilistic rather than absolute. It predicts a broad tendency for recursive self-modeling to be more fragile than integrated first-order experiential organization under perturbation, but it does not require every altered state, clinical condition, species, or experimental task to follow the same sequence. Several boundary conditions are therefore important.

#### 3.4.1 Measurement asymmetry

Apparent exceptions may arise from measurement asymmetry. First-order experiential organization can be difficult to assess when report, motor response, or behavioral access is impaired. In such cases, self-related processing may appear preserved relative to experiential organization because the latter is harder to detect, not because it is absent.

#### 3.4.2 Minimal versus recursive self-related processing

Some self-related processes are minimal rather than fully recursive. Own-name effects, self-prioritization, body ownership, or simple agency attribution may persist where metacognitive monitoring, autobiographical continuity, or temporally extended selfhood is disrupted. Such cases would not necessarily falsify the framework because it distinguishes minimal self-related processing from robust recursive self-modeling.

#### 3.4.3 Pathology-specific dissociations

Pathology-specific dissociations may produce unusual profiles. Some neurological or psychiatric conditions may selectively disrupt perceptual organization, bodily integration, affective ownership, or autobiographical continuity while preserving other forms of verbal report or self-description. These cases may generate apparent inversions of the predicted sequence, but they may also reveal that different components of recursive self-modeling depend on different organizational conditions.

#### 3.4.4 Narrative reconstruction vs. online monitoring

Narrative self-report may sometimes be preserved or reconstructed despite impaired online monitoring. A subject may produce coherent retrospective accounts even where confidence calibration, agency judgment, or metacognitive access was unstable during the state being investigated. This reinforces the need to distinguish *post-hoc* narrative reconstruction from recursive self-modeling during perturbation.

#### 3.4.5 Domain-specific dissociation

Dissociation may be domain-specific. Metacognitive access may remain partly available in one domain while being impaired in another. Confidence monitoring for a simple perceptual task, for example, would not by itself imply preserved autobiographical continuity, agency stability, or temporally extended self-related regulation.

#### 3.4.6 Implications for testing the framework

For these reasons, the framework should not be tested through single markers or isolated behavioral indicators. It would be weakened most clearly if robust recursive self-modeling were consistently preserved across multiple measures despite major disruption to the organizational conditions supporting first-order experiential organization, or if first-order experiential organization and recursive self-modeling consistently degraded and recovered together without meaningful dissociation.

### 3.5 Transition zones rather than fixed boundaries

The framework uses transition-zone language to avoid treating awareness processes as simple binary states or sharply bounded natural kinds.

Awareness processes likely vary across multiple interacting dimensions, including:

- representational coordination,
- the temporal stability of representations, access, and self-related organization across time,
- behavioral flexibility,
- recurrent processing,
- metacognitive accessibility,
- and recursive self-related regulation.

Here, temporal stability refers to the degree to which representations, access relations, experiential contents, and self-related organization remain coordinated across time rather than fragmenting, fluctuating, or rapidly decaying.

For this reason, the proposed transitions should not be interpreted as fixed cut-points. They are better understood as regions of organizational change in which different capacities may emerge, degrade, or recover at different rates.

This approach aligns with multidimensional approaches to consciousness, which caution against reducing conscious states to a single linear scale or binary distinction (Bayne et al., 2016). On the present view, altered states may involve different profiles of preserved and disrupted capacities rather than uniform movement from consciousness to unconsciousness.

This is especially relevant for sleep onset, dreaming, anesthetic induction and emergence, disorders of consciousness, developmental change, dissociation, depersonalization, and other altered states. Such conditions often involve mixed organizational profiles rather than simple presence or absence of consciousness.

The framework therefore predicts multidimensional variation rather than uniform transitions between conscious and unconscious states.

### 3.6 Empirical relevance

The transition-zone model is intended to support empirical investigation rather than to provide a metaphysical account of consciousness. Its main empirical value lies in predicting dissociations between:

- first-order experiential organization,
- behavioral responsiveness,
- metacognitive accessibility,
- agency attribution,
- confidence estimation,
- and temporally extended self-related continuity.

The strongest tests of the framework would examine these dimensions together across changing states. For example, perturbation and recovery paradigms could test whether experiential organization and recursive self-modeling degrade and recover together or show partially ordered dissociation.

The following sections develop the structural dependence underlying this proposal and clarify how awareness processes may vary with organizational constraint, regulation, and recursive accessibility.

## 4 Structural dependence and organizational constraint

Central to the present framework is the principle of structural dependence: awareness processes vary according to underlying organizational conditions. These include representational coordination, temporal stability, constrained access, adaptive regulation, and recursive accessibility.

The framework does not propose that awareness processes can be reduced to informational quantity, behavioral complexity, or signal richness alone. Increasing information processing, adaptive responsiveness, or behavioral sophistication is not assumed to generate integrated experiential awareness or recursive self-modeling unless the relevant organizational conditions are also present.

Instead, the framework proposes that awareness processes depend on how information is accessed, coordinated, stabilized, and incorporated into ongoing behavioral regulation across time.

This principle helps explain why altered states may produce partial dissociations rather than uniform loss of a single consciousness property.

### 4.1 Constrained access

All biological systems operate under conditions of constrained access. Organisms do not access the world in its entirety. They process limited informational subsets shaped by sensory architecture, biological relevance, metabolic demands, environmental pressures, and organizational capacity.

Constrained access is not itself sufficient for integrated experiential awareness. Local predictive control, adaptive responsiveness, and uncertainty-sensitive regulation may occur without stable experiential organization or recursive self-modeling.

Within the present framework, constrained access becomes relevant to awareness processes when available information is coordinated across domains, stabilized across time, and incorporated into flexible behavioral regulation.

This allows the framework to distinguish simple responsiveness from more organized forms of experiential and self-related processing.

### 4.2 Organizational stability and experiential organization

Integrated experiential awareness is proposed to depend on sufficient organizational stability across distributed processes. This includes:

- cross-domain coordination,
- recurrent processing,
- temporal continuity,
- contextual stability,
- and sustained experiential organization.

Importantly, the framework does not treat any single measure as identical with experiential organization itself. Complexity measures, effective connectivity, recurrent dynamics, large-scale integration, behavioral flexibility, and reportability may each provide useful evidence, but none is sufficient on its own.

This distinction is important because altered states may preserve some organizational dimensions while disrupting others.

For example, dreaming may preserve vivid experiential organization while reducing metacognitive accessibility and autobiographical continuity. Similarly, some disorders of consciousness may preserve partial forms of experiential organization despite severely reduced behavioral responsiveness or explicit report.

Examples include the minimally conscious state, in which behavioral evidence of awareness may be inconsistent but reproducible, and cases of cognitive motor dissociation or covert consciousness, in which patients who appear behaviorally unresponsive may nevertheless show task-related neural responses to commands. These cases suggest that behavioral responsiveness, reportability, and awareness-related organization can diverge, supporting the need to distinguish experiential organization from overt behavioral output or explicit report (Giacino et al., 2002; Owen et al., 2006; Monti et al., 2010; Schiff, 2015).

The framework therefore predicts that experiential organization should vary with changing organizational stability rather than appearing or disappearing as a simple binary state.

### 4.3 Recursive accessibility and self-related organization

Recursive self-modeling is proposed to depend on additional organizational demands beyond integrated experiential awareness. These include:

- recursive accessibility to internal states,
- metacognitive monitoring,
- confidence estimation,
- agency attribution,
- temporal continuity,
- and self-related regulation across time.

The framework does not treat these capacities as fully unified or universally co-dependent. Minimal self-related processing, metacognitive monitoring, and extended autobiographical continuity may vary partially independently.

This distinction helps explain why altered states may preserve experiential organization while disrupting self-reference, confidence calibration, agency attribution, or autobiographical continuity.

Under this interpretation, recursive self-modeling is not a separate Cartesian or substance-like self added to experience. Rather, it is treated as an organizational process in which internal states become available for monitoring, evaluation, and regulation across time. This distinction is consistent with the view that selfhood can be analyzed as a structured process of self-modeling rather than as a separate inner entity or metaphysical subject (Metzinger, 2003).

### 4.4 Structural dependence and ordered dissociation

Structural dependence supports the framework's ordered vulnerability hypothesis.

If recursive self-modeling depends on additional organizational conditions beyond integrated experiential awareness, then it should often be more vulnerable under perturbation. It may destabilize earlier, degrade more rapidly, or recover later than first-order experiential organization.

This does not imply a perfectly uniform sequence across all states or systems. Rather, it predicts a broad tendency: processes requiring recursive accessibility, metacognitive monitoring, and temporally extended self-related regulation should often be more fragile than processes supporting first-order experiential organization.

The framework therefore interprets dissociation not as evidence for multiple independent consciousnesses, but as evidence that different awareness processes depend on partially distinct organizational conditions.

### 4.5 Contribution of structural dependence

The principle of structural dependence gives the framework its main explanatory role. The paper does not claim to introduce integration, access, regulation, metacognition, or self-representation as new concepts. These are already central to existing theories of consciousness.

The contribution is the way these dimensions are organized into a dependency model. This model predicts that awareness processes should not always degrade or recover together, because they do not all depend on the same organizational requirements.

In this sense, the framework aims to clarify relationships between existing theoretical constructs while generating a specific empirical prediction: first-order experiential organization and recursive self-modeling should often show partially ordered dissociation across perturbation and recovery.

## 5 Experience and regulation under uncertainty

The present framework proposes that integrated experiential awareness may contribute to specific forms of regulation under uncertainty. This claim is intentionally narrow. It does not imply that all regulation requires awareness, or that awareness exists for a single adaptive purpose.

Many forms of regulation occur without integrated experiential organization or recursive self-modeling. Homeostatic control, autonomic regulation, sensorimotor adjustment, anticipatory physiological change, and predictive regulation may each occur without stable first-order experiential organization.

The proposal developed here is more limited: integrated experiential awareness may support forms of regulation that require globally coordinated, temporally extended, cross-domain organization under uncertainty.

### 5.1 Regulation without awareness

Biological systems can regulate behavior and maintain viability through local and distributed control processes. Such processes may be adaptive, flexible, and uncertainty-sensitive without implying integrated experiential awareness.

Examples include:

- chemotaxis,
- reflexive coordination,
- autonomic regulation,
- tropistic behavior,
- homeostatic control,
- and anticipatory physiological adjustment.

These examples show that regulation under uncertainty is not unique to awareness processes.

Within the present framework, biological regulation therefore remains distinct from integrated experiential awareness. Regulation alone is not sufficient evidence for experiential organization.

### 5.2 Integrated experiential awareness and cross-domain regulation

Integrated experiential awareness may become functionally relevant when multiple informational domains must be coordinated and stabilized across time.

Such conditions may involve:

- sensory information,
- interoceptive states,
- affective significance,
- memory,
- motivational demands,
- environmental uncertainty,
- and possible behavioral outcomes.

The framework proposes that integrated experiential awareness may support behavioral regulation by maintaining coherent first-order experiential organization across these domains.

This may allow systems to compare competing demands, sustain context-sensitive models, and select behavior flexibly across changing conditions.

Importantly, this does not mean that experiential organization guarantees adaptive or rational behavior. Systems may remain biased, maladaptive, unstable, or self-destructive despite coherent experiential organization.

The narrower claim is that integrated experiential awareness may expand the coordination space available for temporally extended, cross-domain regulation under uncertainty.

### 5.3 Recursive self-modeling and metacognitive regulation

Recursive self-modeling may add a further regulatory layer by making aspects of a system's own internal states available for monitoring and evaluation. This may support:

- confidence estimation,
- error monitoring,
- agency attribution,
- self-evaluation,
- behavioral adjustment,
- and regulation relative to temporally extended self-representations.

Under this interpretation, recursive self-modeling does not simply add more behavioral flexibility. It allows a system to regulate behavior partly in relation to its own uncertainty, performance, goals, and self-related continuity across time.

This is why recursive self-modeling is predicted to be more vulnerable under perturbation. It depends not only on first-order experiential organization, but also on recursive access to internal states and their stabilization across time.

### 5.4 Limits of the functional claim

The framework does not attempt to explain why subjective experience exists at all. Nor does it claim that experiential organization can be reduced fully to behavioral function, computational optimization, or adaptive control.

Its claim is organizational and operational: integrated experiential awareness may contribute to globally coordinated, temporally extended regulation under uncertainty, while recursive self-modeling may support metacognitive regulation through confidence estimation, self-evaluation, agency monitoring, and temporally extended self-related organization.

This framing leaves broader metaphysical questions open.

The purpose of the present section is therefore not to identify the function of consciousness as such, but to clarify one possible functional role for integrated experiential awareness and recursive self-modeling within the broader dependency structure proposed by the framework.

## 6 Relationship to existing theories

The present framework is not proposed as a replacement for existing theories of consciousness. Its role is narrower: to provide an organizational crosswalk for distinguishing awareness processes that are often discussed under shared terminology.

Existing theories already explain important dimensions of consciousness research, including informational integration, global accessibility, predictive regulation, metacognition, and self-representation. The present framework attempts to clarify how these dimensions may relate to partially distinct organizational conditions.

Its contribution is therefore not a new mechanism of consciousness, but a structured way of comparing existing theoretical constructs while generating the ordered vulnerability prediction developed in Sections 3 and 4.

### 6.1 Integrated information theory

Integrated Information Theory (IIT) emphasizes integrated and differentiated informational structure within a system (Tononi, 2004; Tononi et al., 2016).

The present framework aligns with IIT in treating integration as an important dimension of experiential organization. Integrated experiential awareness, as defined here, depends partly on coordinated and stabilized information across multiple domains.

However, the present framework differs from IIT by distinguishing integrated experiential organization from recursive self-modeling. On this account, integration may support coherent first-order experiential organization without necessarily producing metacognitive accessibility, confidence monitoring, autobiographical continuity, or explicit self-related evaluation.

The framework therefore treats integration as important but not sufficient for explaining all awareness processes. In particular, it proposes that recursive self-modeling depends on additional organizational conditions involving recursive accessibility to internal, evaluative, and behavioral states across time.

### 6.2 Global workspace and global neuronal workspace approaches

Global Workspace Theory and Global Neuronal Workspace approaches emphasize large-scale accessibility, distributed broadcasting, and flexible behavioral coordination (Baars, 1988; Dehaene and Changeux, 2011).

The present framework aligns with these approaches in treating large-scale access and coordination as important for integrated experiential awareness.

However, the framework does not equate experiential organization solely with reportability or explicit access. It allows that first-order experiential organization may persist under conditions in which verbal report, reflective monitoring, or self-related accessibility are reduced.

The framework also distinguishes global access-like coordination from recursive self-modeling. Recursive self-modeling requires additional organizational conditions involving metacognitive monitoring, confidence estimation, self-evaluation, and temporally extended self-related regulation.

This distinction helps clarify why altered states may preserve experiential organization while disrupting reflective self-monitoring, confidence evaluation, or autobiographical continuity.

### 6.3 Predictive processing and active inference

Predictive processing and active-inference approaches emphasize modeling, prediction, uncertainty reduction, and adaptive regulation (Friston, 2010; Seth et al., 2012).

The present framework aligns with these approaches in treating organisms as constrained systems that regulate behavior under uncertainty.

However, the framework does not treat uncertainty-sensitive regulation as sufficient evidence for awareness. Many biological processes involve prediction, regulation, and adaptive control without necessarily supporting stable first-order experiential organization or recursive self-modeling.

The framework therefore narrows the relevant claim: integrated experiential awareness may contribute to globally coordinated, temporally extended, cross-domain regulation under uncertainty. Recursive self-modeling may add a further layer by supporting confidence estimation, self-evaluation, agency monitoring, and regulation relative to temporally extended self-representations.

In this respect, the framework distinguishes general adaptive regulation from the more specific organizational conditions associated with integrated experiential awareness and recursive self-modeling.

### 6.4 Higher-order, metacognitive, and self-model approaches

Higher-order, metacognitive, and self-model approaches emphasize reflective accessibility, self-representation, confidence monitoring, and the modeling of internal states (Lau and Rosenthal, 2011; Metzinger, 2003).

The present framework aligns most closely with these approaches in its account of recursive self-modeling. It agrees that metacognitive accessibility, confidence estimation, and self-monitoring depend on additional organizational processes beyond first-order experiential organization alone.

However, the framework does not treat higher-order representation as necessary for all experiential organization. Integrated experiential awareness may occur without fully developed metacognitive monitoring, autobiographical continuity, explicit self-reference, or stable narrative selfhood.

The framework also distinguishes between minimal self-related processing, core metacognitive self-monitoring, and extended autobiographical or narrative selfhood. This distinction reduces the risk of treating selfhood as a single all-or-none capacity.

Recursive self-modeling is therefore understood as layered and potentially dissociable across altered states, development, and neurological disruption.

### 6.5 First-order and reflexive accounts

First-order theories of consciousness hold that experiential awareness need not depend on a separate higher-order or reflective representation. On these accounts, a conscious state may be conscious in virtue of its own first-order representational organization rather than because it is represented by another mental state (Dretske, 1995; Tye, 1995). Reflexive and self-representational approaches similarly emphasize that awareness may involve an intrinsic or pre-reflective form of self-presence within experience, rather than an additional act of explicit self-monitoring (Kriegel, 2009; Zahavi, 2005).

The present framework is compatible with this distinction. Integrated experiential awareness is not defined by reflective access, verbal report, or recursive self-modeling. It may therefore include forms of first-order or reflexive experiential organization that do not amount to metacognitive monitoring, autobiographical continuity, or temporally extended self-representation. Within the framework, recursive self-modeling is reserved for cases in which internal, evaluative, or behavioral states become available as objects of monitoring, confidence estimation, agency attribution, or self-related regulation across time.

### 6.6 Organizational crosswalk

Across these theories, the central issue is not whether one account must replace all others. Rather, different theories may emphasize different dimensions of awareness processes.

IIT foregrounds integration. Global Workspace approaches foreground accessibility and broadcasting. Predictive processing and active inference foreground modeling and regulation under uncertainty. Higher-order, metacognitive, and self-model approaches foreground reflective access and self-related representation. First-order and reflexive accounts foreground experiential organization or pre-reflective self-presence without requiring separate higher-order monitoring.

The present framework proposes that these dimensions may be organized around three partially separable conditions:

- biological regulation,
- integrated experiential awareness,
- and recursive self-modeling.

Its contribution is to clarify how these conditions may relate and to generate a specific empirical prediction: recursive self-modeling should often be more vulnerable than integrated experiential organization under perturbation and slower to re-emerge during recovery.

From this perspective, some disagreements within consciousness science may arise not only from incompatible theories, but from attempts to explain different organizational processes under the same conceptual label.

## 7 Selfhood, metacognition, and recursive accessibility

Selfhood is often discussed as though it were a single capacity. In practice, self-related processing includes several partially separable dimensions, including bodily self-relatedness, agency attribution, metacognitive monitoring, confidence estimation, autobiographical continuity, and narrative identity.

The present framework treats these as layered awareness processes rather than as expressions of one indivisible self. This distinction is important because altered states, development, and neurological disruption may affect different self-related capacities in different ways.

Recursive self-modeling refers to the capacity of a system to represent aspects of its own internal, evaluative, or behavioral states across time. It is not treated as a separate metaphysical entity, but as an organizational process involving recursive accessibility, monitoring, and regulation.

### 7.1 Minimal self-related processing

Some self-related processes are closely tied to bodily regulation, action, and immediate organism-environment coordination. Minimal self-related processing refers here to immediate organism-centered forms of self-relevance that may organize experience or behavior around the body or organism without requiring explicit reflection, metacognitive monitoring, autobiographical continuity, or temporally extended narrative selfhood. These may include:

- body ownership,
- interoceptive salience,
- agency attribution,
- self-prioritization,
- and immediate first-person behavioral orientation.

The framework does not treat these capacities as sufficient evidence for full recursive self-modeling. Own-name effects, self-prioritization, mirror recognition, or bodily self-attribution may each provide useful evidence of self-related processing, but none is sufficient on its own to establish robust metacognitive self-modeling or temporally extended selfhood.

Minimal self-related processing is therefore distinguished from both integrated experiential awareness and more developed forms of recursive self-modeling.

### 7.2 Metacognitive self-monitoring

Metacognitive self-monitoring refers to recursive accessibility to aspects of a system's own cognitive or evaluative states.

This may include:

- confidence estimation,
- uncertainty monitoring^1^,
- error awareness,
- performance evaluation,
- and reflective access to ongoing cognitive processes.

Within the present framework, metacognitive self-monitoring is one core component of recursive self-modeling. It allows a system not only to represent the world, but to evaluate aspects of its own representational or behavioral performance.

Metacognitive capacities can be investigated through measures such as confidence–accuracy relationships, metacognitive sensitivity^2^, and metacognitive efficiency (Fleming and Lau, 2014). These measures are relevant because they allow recursive self-monitoring to be studied operationally rather than treated as an all-or-none introspective capacity.

Importantly, metacognitive self-monitoring is not treated as necessary for all experiential organization. Ordinary dreaming, dissociation, depersonalization, and some anesthetic transitions may preserve vivid experiential organization while reducing reflective monitoring, confidence calibration, or explicit self-evaluation.

This supports the framework's central distinction between integrated first-order experiential organization and recursive self-modeling.

### 7.3 Temporally extended self-representation

More extended forms of selfhood involve continuity across time. These may include:

- autobiographical continuity,
- narrative identity,
- future self-projection,
- temporally extended agency,
- and long-term goal regulation.

The framework does not assume that these capacities emerge together or degrade together. Core metacognitive monitoring may be partly preserved while autobiographical continuity is disrupted. Similarly, narrative selfhood may become unstable while aspects of agency attribution or confidence estimation remain partially available.

This distinction helps avoid treating recursive self-modeling as a single all-or-none capacity. Instead, recursive self-modeling is understood as a layered organizational condition involving multiple self-related processes with different stability profiles.

### 7.4 Altered states and self-related dissociation

Altered states provide important evidence that experiential organization and self-related organization may vary partially independently.

Ordinary dreaming may preserve vivid perceptual and affective organization while reducing reflective monitoring and autobiographical continuity. Lucid dreaming may partially restore metacognitive accessibility and agency while preserving the broader experiential structure of the dream state.

Dissociation and depersonalization may preserve perceptual organization while altering bodily ownership, agency attribution, emotional ownership, or narrative continuity. Neurological disruption and disorders of consciousness may also produce uneven changes across behavioral responsiveness, experiential organization, metacognitive accessibility, and self-related continuity.

Within the present framework, such cases are not interpreted as evidence for multiple independent selves or consciousnesses. They are interpreted as evidence that different self-related processes depend on partially distinct organizational conditions.

### 7.5 Recursive accessibility and ordered vulnerability

Recursive self-modeling is predicted to be more vulnerable than integrated experiential awareness because it depends on additional organizational requirements. These include:

- recursive accessibility to internal states,
- metacognitive monitoring,
- temporal continuity,
- confidence estimation,
- agency attribution,
- and self-related regulation across time.

Under perturbation, these capacities may destabilize before first-order experiential organization fully collapses. During recovery, first-order experiential organization may return before stable recursive self-modeling is restored.

This prediction is probabilistic rather than absolute. The framework does not claim that every altered state must show the same ordering. It predicts a broad tendency: self-related and metacognitive processes should often show greater vulnerability than integrated experiential organization under conditions of reduced organizational stability.

### 7.6 Selfhood as organizational process

The framework interprets selfhood as an organizational process rather than a fixed entity or metaphysical substance.

Its contribution is not to settle philosophical debates about the self, but to separate self-related processes that are often compressed together. Minimal self-related processing, metacognitive self-monitoring, and temporally extended selfhood may overlap, but they are not identical.

This distinction supports the broader claim of the paper: awareness processes are not a single indivisible property. They are partially dissociable organizational processes that may degrade, persist, or recover differently across changing biological and cognitive conditions.

## 8 Operationalization and empirical investigation

The present framework is intended to generate empirically investigable predictions rather than function only as a conceptual distinction. Its central prediction is that integrated experiential awareness and recursive self-modeling should sometimes dissociate across changing biological and cognitive states.

The strongest tests of the framework are therefore not single-marker studies, but multi-measure paradigms that examine several dimensions together, including experiential organization, behavioral responsiveness, metacognitive accessibility, confidence estimation, agency attribution, and self-related continuity.

### 8.1 Testable predictions

The framework does not propose any single definitive measure of awareness. Instead, different measures may track different organizational dimensions.

Relevant measures may include:

- signal complexity,
- effective connectivity,
- recurrent coordination,
- behavioral responsiveness,
- content-sensitive behavior,
- confidence calibration,
- metacognitive sensitivity,
- agency judgments,
- and self-related report.

These measures should not be treated as interchangeable. Complexity, reportability, behavioral flexibility, and metacognitive performance may each provide useful evidence, but none is sufficient on its own to establish the full organizational condition being investigated.

For example, measures of confidence calibration and metacognitive sensitivity may help assess recursive self-monitoring, but they should not be treated as equivalent to experiential organization itself (Fleming and Lau, 2014). Similarly, measures of complexity or effective connectivity may provide evidence relevant to integrated experiential awareness without functioning as direct measurements of awareness as such.

This distinction is important because the framework predicts that these measures may dissociate under altered states rather than rising and falling together. A perturbation may reduce metacognitive accessibility while preserving aspects of experiential organization, or disrupt reportability while leaving some content-sensitive processing intact.

The aim is therefore not to identify one master marker of consciousness, but to examine how different organizational measures vary together or dissociate across changing biological and cognitive states.

### 8.2 Perturbation and recovery paradigms

The most direct way to test the ordered vulnerability hypothesis is through perturbation and recovery paradigms. These include:

- anesthetic induction and emergence,
- sleep onset and waking,
- REM dreaming and lucid dreaming,
- pharmacological perturbation,
- disorders of consciousness,
- and longitudinal recovery from neurological disruption.

The framework predicts that recursive self-modeling should often show greater vulnerability than integrated experiential awareness under these conditions. This predicted pattern of partially dissociable degradation and recovery is represented schematically in Figure 2. In practical terms, confidence calibration, agency attribution, reflective self-monitoring, and autobiographical continuity may degrade while aspects of experiential organization remain partially preserved.

During recovery, the reverse pattern may occur. First-order experiential organization may return before stable metacognitive accessibility or temporally extended self-related continuity is restored.

This prediction is probabilistic rather than absolute. The framework does not require every altered state to show the same sequence. It predicts a broad tendency across sufficiently informative perturbation and recovery contexts.

To illustrate how the ordered vulnerability hypothesis might be operationalized empirically, we provide a hypothetical example using anesthetic induction and recovery.

### 8.3 Worked example: anesthetic induction and recovery

The following example is illustrative rather than prescriptive. It is intended to show how the framework could guide a multivariable perturbation and recovery design.

#### 8.3.1 Context

A study could examine participants undergoing clinically indicated general anesthesia, or controlled sedation where ethically appropriate. The design would require repeated measurement across changing states rather than comparison between wakefulness and anesthesia alone.

#### 8.3.2 Timepoints

Measures could be collected during baseline wakefulness, light sedation, loss of behavioral responsiveness, stable anesthesia, early emergence, and post-recovery orientation. This would allow the trajectory of different awareness-related processes to be compared across both degradation and recovery.

#### 8.3.3 Measures of integrated experiential organization

Candidate measures could include signal complexity, effective connectivity, recurrent coordination, content-sensitive behavioral response where possible, auditory or visual discrimination tasks, and post-recovery reports of dream-like, fragmented, or preserved experience. These measures would be interpreted as evidence relevant to first-order experiential organization, not as direct or sufficient measures of experience itself.

#### 8.3.4 Measures of recursive self-modeling

Candidate measures could include confidence ratings, confidence–accuracy relationships, metacognitive sensitivity, agency judgment, self-location or body-orientation reports, autobiographical orientation, and the capacity to evaluate one's own perceptual or cognitive state during emergence.

#### 8.3.5 Predicted pattern

Consistent with the general pattern illustrated in Figure 2, the ordered vulnerability hypothesis predicts that recursive self-modeling measures should often decline earlier or more steeply than measures associated with first-order experiential organization. During emergence, first-order experiential organization may return before stable confidence calibration, autobiographical orientation, agency judgment, or explicit self-evaluation.

For example, a participant may later report dream-like or fragmented experience while still lacking reliable metacognitive monitoring or temporally extended self-orientation during early recovery.

#### 8.3.6 Disconfirming pattern

Such a design would support the framework if experiential organization and recursive self-modeling showed graded, partially dissociable trajectories across induction and recovery. It would weaken the framework if these dimensions consistently rose and fell together, or if robust recursive self-modeling remained preserved across multiple measures despite substantial disruption to first-order experiential organization.

### 8.4 Sleep and dreaming

Anesthesia and sleep provide particularly relevant domains because they involve dynamic changes in responsiveness, reportability, experiential organization, and self-related processing.

Under anesthesia, the framework predicts that measures associated with first-order experiential organization and measures associated with recursive self-modeling may degrade and recover at different rates. For example, behavioral responsiveness and report may not perfectly track experiential organization, while confidence estimation, agency monitoring, and autobiographical continuity may show additional vulnerability during induction or emergence.

Dreaming provides a complementary domain. Ordinary dreams may preserve vivid experiential organization while reducing reflective monitoring, stable agency, and autobiographical continuity. Lucid dreaming may partially restore metacognitive accessibility and agency while retaining the broader experiential structure of the dream state.

These cases are not treated as proof of the framework. They are proposed as test domains in which the predicted dissociation can be investigated.

### 8.5 Disorders of consciousness

Disorders of consciousness provide another domain in which behavioral responsiveness, experiential organization, and self-related accessibility may diverge.

The framework predicts that some cases may show evidence consistent with preserved or recovering first-order organization despite limited behavioral report or unstable self-related processing. This makes it important to avoid treating overt responsiveness as the only indicator of relevant awareness processes.

Developmental trajectories may also be informative. The framework predicts that flexible experiential engagement and cross-domain coordination should generally emerge before robust metacognitive calibration, autobiographical continuity, and temporally extended self-representation.

This prediction should not be read as a rigid developmental stage theory. It is a broad organizational expectation: recursive self-modeling should depend on additional conditions beyond first-order experiential organization.

### 8.6 Comparative cognition and boundary cases

Comparative cognition and artificial systems provide useful boundary cases for the framework, but they should be treated cautiously. The framework does not infer awareness processes from behavioral sophistication alone. Adaptive behavior, predictive control, flexible problem-solving, or linguistic output are not sufficient evidence for integrated experiential awareness or recursive self-modeling.

Instead, comparative evaluation should focus on organizational profiles: which forms of access, coordination, temporal stability, regulation, and recursive accessibility appear to be present, and which remain uncertain.

In non-human species, biological regulation may be indicated by homeostatic control, reflexive coordination, sensorimotor adaptation, predictive foraging, basic learning, or uncertainty-sensitive behavioral adjustment. These capacities may be highly adaptive without implying integrated experiential organization. They therefore remain evidence of biological regulation unless accompanied by stronger indicators of cross-domain coordination and temporal stability.

Integrated experiential organization would require evidence of more than local responsiveness. Candidate markers may include sustained multimodal integration, flexible context-sensitive behavior, stable world/body modeling, affective and interoceptive coordination, and behavioral selection that is not reducible to immediate stimulus-response coupling. Species such as corvids, primates, cetaceans, and cephalopods may provide useful comparative cases because they show complex forms of flexible action, problem solving, social coordination, or environmental modeling. However, such capacities should be interpreted as evidence relevant to integrated experiential organization, not as decisive proof of experience itself.

Recursive self-modeling would require still more specific evidence. Candidate markers may include uncertainty monitoring, confidence-sensitive decision-making, opt-out behavior in difficult trials, error correction based on self-monitoring, agency discrimination, metacognitive sensitivity, and temporally extended self-related regulation. Mirror recognition, own-name effects, self-prioritization, or episodic-like memory may be relevant, but none is sufficient on its own. Within the present framework, these behaviors must be interpreted in relation to whether the system appears able to monitor, evaluate, or regulate its own internal or behavioral states across time.

No single marker should be treated as decisive; organizational profiles should be evaluated through converging evidence across multiple domains. A compact way to distinguish these domains is shown in Table 2.

**Table 2:** Comparative organizational markers and interpretive cautions.

Organizational condition	Candidate comparative markers	Interpretation caution
Biological regulation	Homeostasis, reflexive coordination, sensorimotor adaptation, predictive foraging, basic learning, uncertainty-sensitive adjustment	Adaptive responsiveness alone is not sufficient evidence for integrated experiential organization
Integrated experiential organization	Cross-modal integration, sustained perceptual organization, flexible context-sensitive action, stable world/body modeling, affective and interoceptive coordination	Behavioral flexibility alone does not establish experience; multiple converging indicators are required
Recursive self-modeling	Confidence-sensitive behavior, opt-out responses, metacognitive sensitivity, agency discrimination, error monitoring, temporally extended self-related regulation	Minimal self-related markers do not establish robust recursive self-modeling or narrative selfhood

The same caution applies to artificial systems. The framework remains agnostic about whether non-biological systems can satisfy the relevant organizational conditions. Linguistic fluency, behavioral sophistication, or flexible problem-solving should not be treated as sufficient evidence for integrated experiential awareness or recursive self-modeling. The relevant question is whether a system exhibits the organizational dependencies identified by the framework: constrained access, representational coordination, temporal stability, adaptive regulation, and recursive accessibility to its own internal or behavioral states.

The aim is therefore not to rank species or systems on a single scale of consciousness. It is to ask which awareness-related processes, if any, are supported by the organizational profile of a given system, and where biological regulation, integrated experiential organization, and recursive self-modeling may dissociate.

### 8.7 Toward a multidimensional research program

The framework supports a multidimensional approach to consciousness research. Rather than seeking a single marker for consciousness as such, it encourages investigation of how different awareness processes relate across changing states.

The most informative studies would jointly measure:

- first-order experiential organization,
- behavioral responsiveness,
- metacognitive accessibility,
- confidence estimation,
- agency attribution,
- and self-related continuity.

The framework would be supported if recursive self-modeling frequently shows greater vulnerability than integrated experiential awareness under perturbation and slower restoration during recovery.

It would be weakened if these processes consistently rise and fall together without meaningful dissociation, or if recursive self-modeling remains robust despite major disruption to first-order experiential organization.

The aim is therefore not to prove a complete theory of consciousness, but to provide a testable organizational framework for examining how different awareness processes degrade, persist, and recover across biological and cognitive states.

## 9 Conclusion

This paper has proposed an organizational framework for distinguishing awareness processes that are often compressed under broad terms such as consciousness, awareness, reportability, selfhood, and metacognition.

The framework distinguishes three related but partially separable organizational conditions:

- biological regulation,
- integrated experiential awareness,
- and recursive self-modeling.

These conditions are not proposed as rigid stages or sharp natural boundaries. They are treated as graded organizational transition zones involving different demands on constrained access, representational coordination, temporal stability, adaptive regulation, and recursive accessibility.

The central empirical claim is an ordered vulnerability hypothesis: recursive self-modeling should often be more vulnerable than integrated first-order experiential organization under perturbation and should often re-emerge later during recovery.

This prediction does not imply that all altered states will follow a single uniform sequence. Rather, it proposes a broad organizational tendency: processes requiring metacognitive accessibility, confidence estimation, agency attribution, and temporally extended self-related continuity should often be more fragile than processes supporting first-order experiential organization.

The framework is not intended as a replacement for existing theories of consciousness. Its contribution is more limited: it provides an organizational crosswalk for relating existing constructs and identifying where different theories may be addressing different awareness processes.

By distinguishing biological regulation, integrated experiential awareness, and recursive self-modeling, the framework aims to support a more precise empirical program. Rather than asking whether consciousness is simply present or absent, researchers may ask which awareness processes are preserved, degraded, dissociated, or restored under changing biological and cognitive conditions.

Progress in consciousness science may therefore depend not only on identifying neural correlates or theoretical mechanisms, but also on clarifying the organizational relationships between first-order experiential organization, behavioral regulation, metacognitive accessibility, and recursive self-related processing.

## Data Availability

No datasets were generated or analyzed for this study.

## Footnotes

1 Uncertainty monitoring refers here to a system's capacity to track the reliability, confidence, or ambiguity of its own perceptual, cognitive, or behavioral states. Its function is to support adjustment when information is incomplete, unstable, or unreliable, such as withholding a response, seeking further information, or modifying subsequent behavior.

2 Metacognitive sensitivity refers to the degree to which confidence judgments distinguish between correct and incorrect first-order performance. It is narrower than general metacognition, which refers more broadly to the monitoring and control of cognitive processes. In this context, metacognitive sensitivity is relevant because it provides an operational measure of how accurately a system can evaluate its own perceptual or cognitive performance.

## References

[B1] Baars B. J. (1988). A Cognitive Theory of Consciousness. New York, NY: Cambridge University Press.

[B2] Bayne T. Hohwy J. Owen A. M. (2016). Are there levels of consciousness? Trends Cognit. Sci. 20, 405–413. doi: 10.1016/j.tics.2016.03.009 27101880

[B3] Dehaene S. Changeux J.-P. (2011). Experimental and theoretical approaches to conscious processing. Neuron 70, 200–227. doi: 10.1016/j.neuron.2011.03.018 21521609

[B4] Dretske F. (1995). Naturalizing the Mind. Cambridge, MA: MIT Press. doi: 10.7551/mitpress/4872.001.0001

[B5] Fleming S. M. Lau H. C. (2014). How to measure metacognition. Front. Hum. Neurosci. 8:443. doi: 10.3389/fnhum.2014.00443 25076880 PMC4097944

[B6] Friston K. (2010). The free-energy principle: A unified brain theory? Nat. Rev. Neurosci. 11, 127–138. doi: 10.1038/nrn2787 20068583

[B7] Giacino J. T. Ashwal S. Childs N. Cranford R. Jennett B. Katz D. I. Kelly J. P. Rosenberg J. H. Whyte J. Zafonte R. D. Zasler N. D. (2002). The minimally conscious state: Definition and diagnostic criteria. Neurology 58, 349–353. doi: 10.1212/WNL.58.3.349 11839831

[B8] Kriegel U. (2009). Subjective Consciousness: A Self-Representational Theory. Oxford: Oxford University Press. doi: 10.1093/acprof:oso/9780199570355.001.0001

[B9] Lau H. Rosenthal D. (2011). Empirical support for higher-order theories of conscious awareness. Trends Cognit. Sci. 15, 365–373. doi: 10.1016/j.tics.2011.05.009 21737339

[B10] Lozito S. Lasaponara S. Liu J. Navarro V. Lehongre K. Frazzini V. Seidel Malkinson T. Doricchi F. Bartolomeo P. (2026). Towards a bridge between intracerebral and surface EEG signatures of conscious report. Neurosci. Conscious. 2026:niag011. doi: 10.1093/nc/niag011 41969663 PMC13069880

[B11] Metzinger T. (2003). Being No One: The Self-Model Theory of Subjectivity. Cambridge, MA: MIT Press. doi: 10.7551/mitpress/1551.001.0001

[B12] Monti M. M. Vanhaudenhuyse A. Coleman M. R. Boly M. Pickard J. D. Tshibanda L. Owen A. M. Laureys S. (2010). Willful modulation of brain activity in disorders of consciousness. New Engl. J. Med. 362, 579–589. doi: 10.1056/NEJMoa0905370 20130250

[B13] Owen A. M. Coleman M. R. Boly M. Davis M. H. Laureys S. Pickard J. D. (2006). Detecting awareness in the vegetative state. Science 313:1402. doi: 10.1126/science.1130197 16959998

[B14] Schiff N. D. (2015). Cognitive motor dissociation following severe brain injuries. JAMA Neurol. 72, 1413–1415. doi: 10.1001/jamaneurol.2015.2899 26502348

[B15] Seth A. K. Suzuki K. Critchley H. D. (2012). An interoceptive predictive coding model of conscious presence. Front. Psychol. 2:395. doi: 10.3389/fpsyg.2011.00395 22291673 PMC3254200

[B16] Tononi G. (2004). An information integration theory of consciousness. BMC Neurosci. 5:42. doi: 10.1186/1471-2202-5-42 15522121 PMC543470

[B17] Tononi G. Boly M. Massimini M. Koch C. (2016). Integrated information theory: From consciousness to its physical substrate. Nat. Rev. Neurosci. 17, 450–461. doi: 10.1038/nrn.2016.44 27225071

[B18] Tye M. (1995). ITen *Problems of Consciousness: A Representational Theory of the Phenomenal Mind*. Cambridge, MA: MIT Press. doi: 10.7551/mitpress/6712.001.0001

[B19] Zahavi D. (2005). Subjectivity and Selfhood: Investigating the First-Person Perspective. Cambridge, MA: MIT Press. doi: 10.7551/mitpress/6541.001.0001

